# Nasojejunal tube‐assisted endoscopic ultrasound‐guided gastrojejunostomy for the management of gastric outlet obstruction is safe and effective

**DOI:** 10.1002/deo2.210

**Published:** 2023-01-30

**Authors:** Praveer Rai, Pankaj Kumar, Amit Goel, Thakur Prashant Singh, Malay Sharma

**Affiliations:** ^1^ Department of Gastroenterology Sanjay Gandhi Postgraduate Institute of Medical Sciences Lucknow India; ^2^ Department of Gastroenterology Aryavrat Hospital Meerut India

**Keywords:** endoscopic ultrasound, gastroenterostomy, gastrojejunostomy, lumen apposing metal stent, therapeutic endoscopic ultrasound

## Abstract

**Background and aims:**

Endoscopic ultrasound‐guided gastrojejunostomy (EUS‐GJ) is a therapeutic option for patients with gastric outlet obstruction (GOO), which provides long‐term luminal patency without the risk of tumor ingrowth and/or overgrowth and avoids surgical morbidity. The goal of this study was to assess technical success, clinical success, and adverse events associated with a nasojejunal tube‐assisted EUS‐ GJ technique.

**Methods:**

This was a retrospective study conducted at a single tertiary care center. The nasojejunal tube (14F) was used to perform the EUS‐GJ (device‐assisted method). During the study period, consecutive GOO patients who underwent EUS‐GJ between August 2018 and December 2021 were included. Technical success was defined as adequate positioning and deployment of the stent. The patient's ability to tolerate a normal oral diet without vomiting was defined as clinical success.

**Results:**

Thirty patients underwent EUS‐GJ during this study period. Twenty‐six patients had malignant GOO, while four had a benign obstruction. EUS‐GJ was successfully performed in 29 patients, and technical success was 96.67% (29/30). Nasojejunal tube‐assisted EUS‐GJ technique was used in all patients. Clinical success was achieved in all patients who had technical success (29/29, 100%). The adverse events rate was 6.6%. During the procedure, the median procedure time was 25 min (interquartile range 15–42.5), and the average hospitalization was 4.4 days. Normal meals were tolerated by all patients. After 210 days of median follow‐up (range 5–880 days), no recurrence of symptoms was observed.

**Conclusion:**

The nasojejunal tube‐assisted EUS‐GJ is a safe and effective technique to treat GOO symptoms.

## INTRODUCTION

Gastric outlet obstruction (GOO) presents with recurrent vomiting and needs intervention to sustain life. For GOO, benign causes have become less prevalent after Helicobacter Pylori was discovered and proton pump inhibitors were more widely used.^1^ Malignant causes of GOO include periampullary neoplasm (e.g., carcinoma head of the pancreas, duodenum, distal bile duct, or the ampulla) or gastric cancer, and these patients commonly experience symptoms such as postprandial vomiting, pain abdomen and an inability to tolerate oral intake.^2^

The traditional treatment for GOO has been surgery and endoscopic enteral stenting or endoscopic balloon dilatation. Surgical intervention is feasible only for a few patients, due to poor general conditions secondary to underlying malignancy and carries the risk of delayed gastric emptying, gastroparesis, prolonged hospitalization, and death.^3^, ^4^ Endoscopic placement of self‐expanding metal stent (SEMS) has fewer risks and requires shorter hospitalization time; however, there are issues concerning long‐term patency as well as recurrence rates, that might require reintervention.^5^ Endoscopic balloon dilation is primarily done for benign causes of GOO like caustic injury or chronic pancreatitis and the outcome is quite variable.

Endoscopic ultrasound‐guided gastrojejunostomy (EUS‐GJ) is an emerging approach to relieve GOO in which a new fistulous tract is created between the stomach and jejunum. In comparison to surgery, EUS‐GJ is less invasive and has lower morbidity. Compared to endoscopic balloon dilatation and endoscopic enteral stenting, EUS‐GJ has long‐term stent patency and a lower risk of recurrence of obstruction.^6^ In addition, EUS‐GJ can be used to treat afferent loop disease.^7^ In EUS‐GJ a biflanged lumen apposing metal stent (LAMS) is used to create the endoscopic anastomosis between the small intestine and stomach. Three different techniques are used in the EUS‐GJ procedure, the first direct unassisted, the second device assisted by a dilation balloon or stone extraction balloon catheter, a nasobiliary drainage catheter, and an ultraslim endoscope. Third, EUS‐guided double‐balloon occluded gastrojejunostomy bypass (EPASS) using a standard double‐balloon enteric tube (Tokyo Medical University type; Create Medic Co., Ltd., Yokohama, Japan), a standard procedure.^8^ While many techniques have been developed and improved for EUS‐GJ, we still don't know which technique is the best for success. EPASS technique is considered to be safe and reliable, however, the device used in this is not commercially available, hence we used the nasojejunal tube which is easily available, inexpensive, and provides adequate distension of the small bowel to perform EUS‐GJ. The objective of this study is to report our experience of performing EUS‐GJ using a nasojejunal tube.

## PATIENTS AND METHODS

This was a retrospective study conducted in a single tertiary care center. We retrospectively reviewed the prospectively collected database of the 30 adult (>18 years of age) patients who underwent EUS‐GJ between August 2018 and December 2021 in our hospital. Clinical symptoms, laboratory tests, and imaging were used to diagnose malignant GOO (including ultrasound abdomen, computerized tomography scan of the abdomen, and upper gastroduodenoscopy). Patients over the age of 18 diagnosed with GOO using the GOO scoring system (GOOSS) were included in the study. The severity of GOO was recorded according to the GOOSS, which grades GOO from 0 to 3 (0: no oral intake, 1: liquid intake only, 2: semi‐solid intake, 3: indicates low residue or full diet).^9^ Gastric body neoplasm, complete GOOs that could not be negotiated with a guidewire, patients with poor performance status (ECOG 3 or more), and gross ascites were excluded. Each patient's medical and endoscopic records, along with the GOO etiology, hospital stay length, procedure‐related adverse events (AEs), clinical success rate, technical success rate, and GOOSS before treatment were also reviewed. Food intake was measured using the GOOSS. The technical success of the procedure was defined as adequate positioning and deployment of the stent. The clinical success of the intervention was determined by the ability of the patient to eat without vomiting within 5 days. The American Society of Gastrointestinal Endoscopy lexicon classification was used to define AEs.^10^ After the procedure, participants were followed until the research came to an end or the death of the patients. Every month, patients were contacted by phone to inquire about symptoms and document symptoms such as recurrences and GOOSS scores following the procedure. Ethical clearance and waiver of consent for this study protocol were given by the institutional ethical committee (IEC code 2022‐01‐DM‐EXP‐45).

## PROCEDURE DETAILS

**FIGURE 1:**
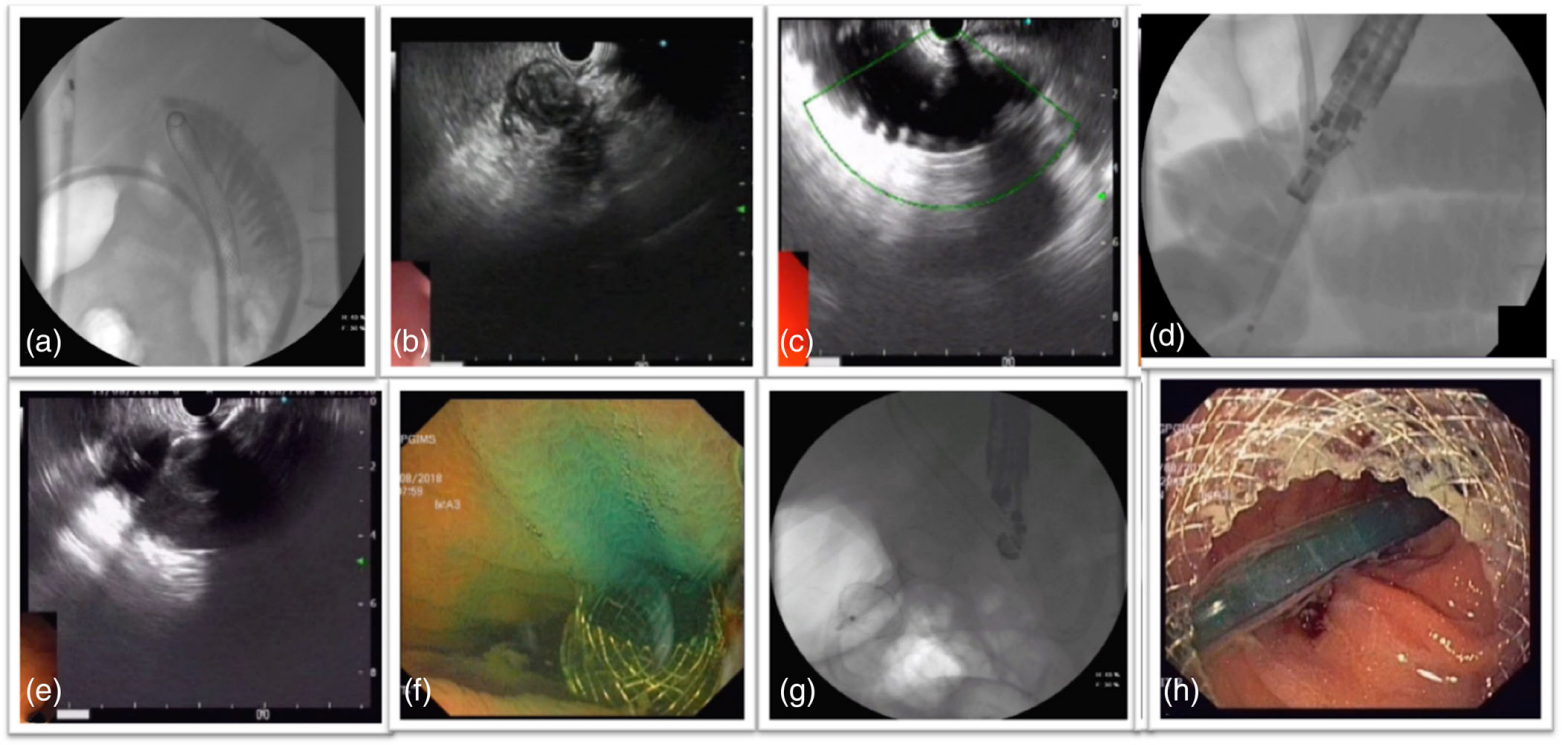
(a) Placement of a nasojejunal tube. (b) Collapsed small bowel loop seen on endoscopic ultrasound. (c) Distension of small bowel loop with normal saline with methylene blue and contrast. (d) Direct puncture of the small bowel loop with Hot Axios stent. (e) Release of the distal flange of Hot Axios stent. (f) Release of the proximal flange of the stent with blue‐colored fluid seen in the stomach. (g) Stent with a waist seen immediately after deployment. (h) Small bowel loop seen with the endoscope

Patients were kept nil orally with continuous nasogastric tube drainage for 48 h before the procedure and received antibiotics. All the procedures were performed by a single experienced endosonographer (Praveer Rai, experience of more than 500 EUS‐guided interventions) using the techniques reported by Itoi et al. and Khashab et al.^11^, ^12^ EUS‐GJ was performed using a device‐assisted technique to construct a gastroenterostomy bypass (Figure 1 and Video S1). First, an upper GI endoscope was used to pass a stiff guidewire in the small intestinal loop distal to the site of obstruction. Once the guidewire was in the desired location the endoscope was withdrawn and the nasojejunal tube was advanced over the wire under fluoroscopic vision. The contrast was administered into the nasojejunal tube to ensure that it was positioned with the desired bowel loop. The guidewire was removed from the patient and the nasojejunal tube was left in situ, with the tip positioned beyond the GOO. During EUS‐GJ, a foot‐pedal‐activated irrigation pump was connected to the nasojejunal tube for fluid infusion. For EUS‐guided access, using an irrigation pump provides a more secure target loop by allowing us to continuously infuse large amounts of fluid distal to the obstruction. The fluid used was normal saline mixed with methylene blue and contrast. By adding normal saline instead of sterile water, hyponatremia caused by dilution can be prevented. Methylene blue is added to saline for visual confirmation of successful LAMS placement while contrast is mixed with saline to fluoroscopically delineate the small bowel. To better distend the distal duodenum and jejunum downstream, approximately 500 ml of fluid is used. A large amount of fluid should be avoided as it distends not only the targeted small intestine but also the colon, leading to mispunctures like gastrocolonostomy. A dose of 20 mg of hyoscine is given intravenously to reduce intestinal peristalsis and prevent the filling of colonic loops. During the time of filling the target jejunal loop, a linear echoendoscope was passed into the stomach. Before puncturing, fluoroscopy and linear echoendoscope were used to identify the distended jejunal segment. A 20 mm LAMS (Massachusetts, Marlborough, Boston Scientific, HOT Axios) was used to perform the EUS‐GJ procedure. The cautery enhanced assembly of the hot Axios stent was passed through the channel of the EUS scope and luer locked on the scope. Using pure cut current, the cautery‐enhanced tip was used to puncture the distal small bowel loop across the stomach wall. The stent's distal flange was then deployed under fluoroscopic and EUS guidance and then under endoscopic guidance, the proximal flange was deployed. The presence of a blue‐colored fluid (due to methylene blue in the instilled normal saline) in the stomach lumen after LAMS deployment suggests that the stent has been placed correctly positioned. After the stent deployment balloon dilation of the stent was not done as the stent opens up fully within 24–48 h and to avoid any risk of migration immediately after deployment. On the day of the procedure, the patients were kept nil orally. At 48 h, an oral contrast study was done to confirm the gastric emptying pathway. After the contrast study, oral liquids were allowed, and a low‐residue diet was allowed the next day. Another three days of antibiotics were prescribed. Patients were discharged on the third post‐procedure day when they were able to eat an oral semisolid meal and were clinically stable. All aspects of the study were evaluated, including clinical success, technical success, and AE. Every case was followed up on monthly basis, either personally or by phone, until death or the last contact.

## STATISTICAL ANALYSIS

Categorical data are expressed as a number, proportion, and ratio whereas continuous data are given as median (interquartile range). Data were compared using non‐parametric tests (Mann‐Whitney U‐test) of significance. The data were analyzed using SPSS, version 23. The level of significance was taken at a *p*‐value < 0.05. The Kaplan‐Meier curve was used for the survival analysis of the patients.

## RESULTS

**FIGURE 2:**
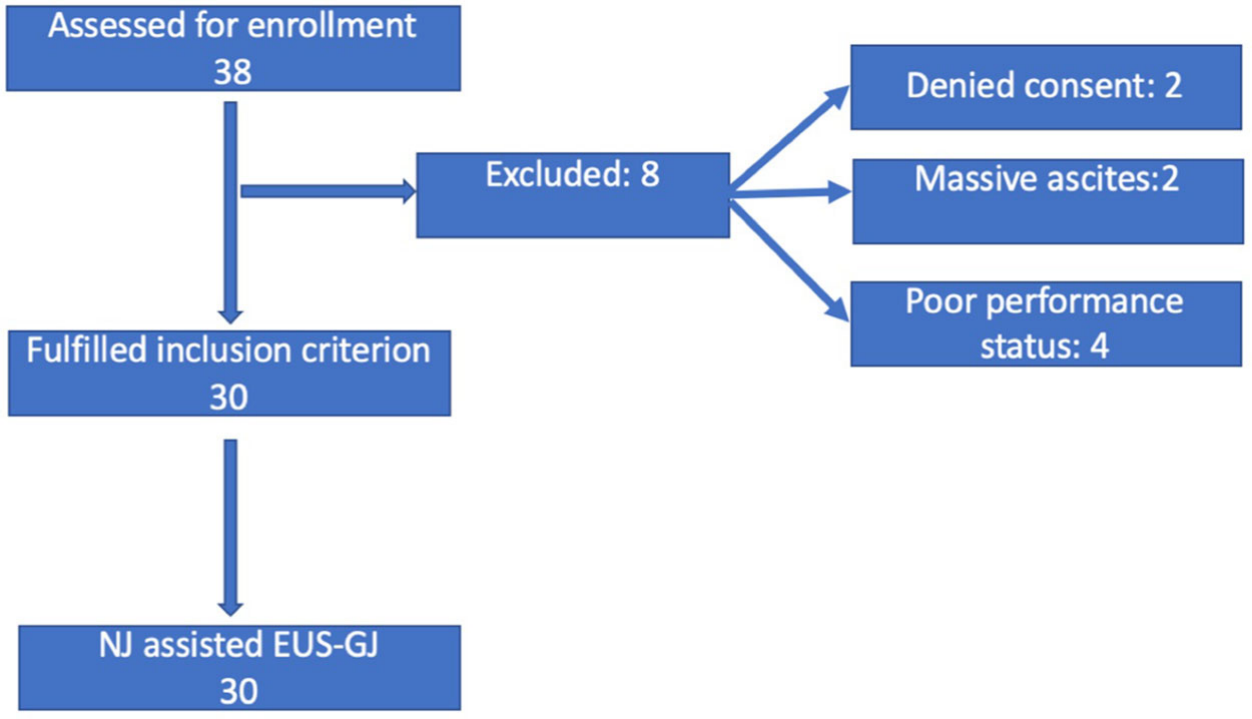
Flow chart of the study

**FIGURE 3:**
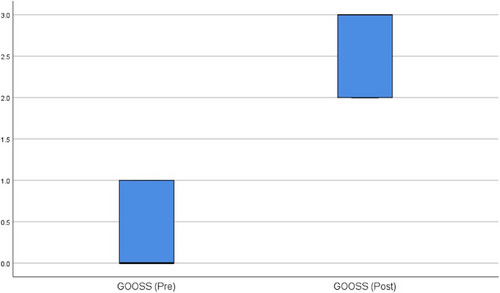
Comparison box plot diagram showing the change in median gastric outlet obstruction scoring system (GOOSS) before and 15 days after the procedure

**FIGURE 4:**
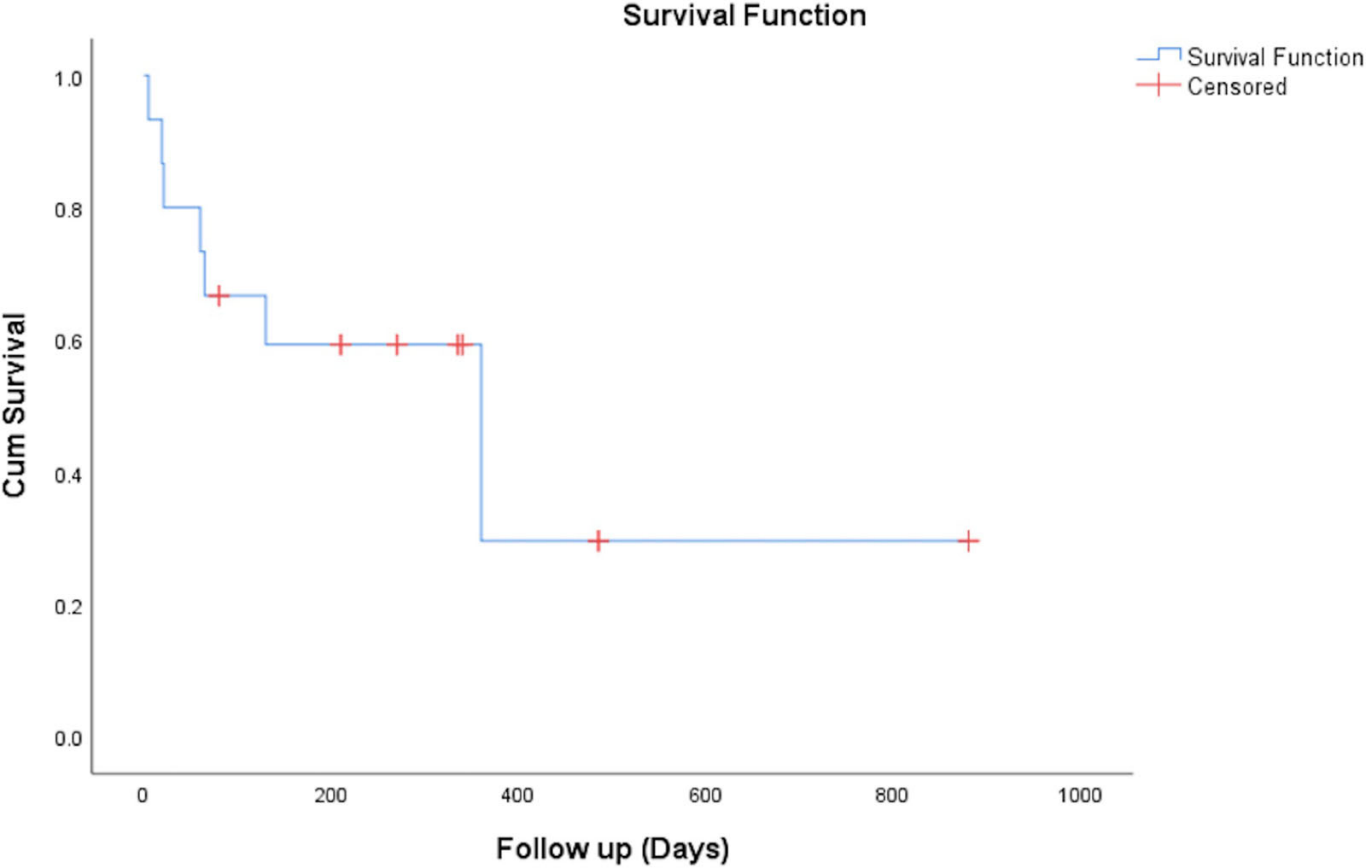
Kaplan Meier analysis showing survival time of gastric outlet obstruction patients

The characteristics of the 30 patients who underwent EUS‐GJ are summarized in Table 1. Figure 2 shows the study flow chart. The GOO was secondary to malignant etiology in the majority (26/30, 86.67%) and carcinoma gall bladder was the most common cause. All patients had vomiting and oral feed intolerance. The second part of the duodenum was the most frequent obstruction site (20/30, 66.67). None of the participants had undergone prior enteral stenting. The outcome of the 30 patients who underwent EUS‐GJ is summarized in Table 2. All patients underwent device‐assisted EUS‐GJ using 14 French nasojejunal tubes. The gastric body posterior wall was punctured in all 30 patients (100%). During the procedure, the median procedure time was 25 min (interquartile range 15–42.5). The technical success rate was 96.67% (29/30). The clinical success rate was 100 % (29/29). During the procedure, there was no bleeding episode. There was no peritonitis or delayed bleeding in any patients. During the procedure, one patient had a stent misdeployment, distal end in the peritoneum and proximal in the stomach, the stent was removed and enteral SEMS was placed without any complication. Ten months after EUS‐GJ, one patient had stent migration, however, the anastomotic site was patent and an enteral SEMS was successfully placed through the GJ fistula. The median GOOSS was significantly improved from 0 before the procedure to 3 in 15 days after the procedure (*p* < 0.0001; Figure 3). After EUS‐GJ, food intake significantly improved. The average length of hospitalization during the procedure was 4.4 days. The survival curve of the patient who underwent EUS‐GJ is shown in Figure 4, and the median survival was 360 days. After a median follow‐up of 210 days (range 5–880 days), none had a recurrence of GOO.

**TABLE 1:** Characteristics of the study participants

Age (years)	59.14 ± 10.58
Sex male, *n* (%)	10 (33.33)
Etiology of obstruction, *n* (%) Gall bladder cancer Gastric cancer Duodenal cancer Gastric lymphoma Pancreatic cancer Groove pancreatitis Post radiotherapy GOO	10 (33.33) 6 (20) 6 (20) 2 (6.66) 2 (6.66) 2 (6.66) 2 (6.66)
Site of obstruction, *n* (%) 2nd part of the duodenum Pylorus	20 (66.67) 10 (33.33)
Prior enteral stenting	None
Biliary obstruction	15 (50)
Median GOOSS (Pre)	0 (0‐1)

Numerical, categorical, and ordinal data are presented as mean (SD), number (%), and median (interquartile range), respectively.

Abbreviations: GOO, gastric outlet obstruction; GOOSS, gastric outlet obstruction scoring system.

**TABLE 2:** Technical details and outcome of the participants who underwent endoscopic ultrasound‐guided gastrojejunostomy (EUS‐GJ)

**Site of puncture**	***N* (%)**
Posterior wall of stomach	30 (100)
Median procedure time (minutes)	25 (15–42.5)
Technical success	29 (96.67)
Clinical success	29 (100)
Median follow up	210 (5–880 days)
Median survival (days)	360
Median GOOSS (Post)	3 (2–3)
EUS‐GJ technique	*n* (%)
Nasojejunal assisted	30 (100)
Adverse event during the procedure Bleeding Stent misdeployment	*n* (%) 0 1 (3.3)
Adverse event after the procedure Stent migration Peritonitis Delayed bleeding	*n* (%) 1 (3.3) 0 0

Numerical, categorical, and ordinal data are presented as mean (SD), number (%), and median (interquartile range), respectively.

Abbreviations: EUS‐GJ, endoscopic ultrasound‐guided gastrojejunostomy; GOOSS, gastric outlet obstruction scoring system.

## DISCUSSION

Surgical gastro‐jejunostomy has long‐term patency and is the most preferred palliative treatment for malignant GOO. Considering its less invasive nature, endoscopic enteral SEMS provides an alternative for surgically ineligible patients.^5^ Due to tumor ingrowth, GOO recurrence is a major drawback of enteral SEMS. Malignant GOO has been treated with EUS‐GJ as a palliative treatment, in recent years. The EUS‐GJ is equivalent to a surgically created fistula between the stomach and jejunum. In contrast to the endoscopic SEMS placement or dilation, the EUS‐GJ creates a bypass at a site away from the site of the lesion and hence remains patent for a relatively longer duration.

There are three types of techniques for performing EUS‐ GJ: [1] direct unassisted, [2] device assisted using a dilatation balloon or stone extraction balloon catheter, Nasobiliary drainage catheter, and ultraslim endoscope, and [3] EUS‐guided double‐balloon occluded gastrojejunostomy bypass (EPASS technique) using a standard double‐balloon enteric tube (Tokyo Medical University type; Create Medic Co., Ltd, Yokohama, Japan), a standard technique.^8^ While many techniques have been developed and improved for EUS‐GJ, we still don't know which technique is the best for success. This study used EUS‐GJ for both malignant and benign GOO. A total of 30 patients had GOO with carcinoma gall bladder as the most prevalent cause, followed by carcinoma stomach. Device‐assisted EUS‐GJ was performed in all patients using a nasojejunal tube (14F). The posterior wall of the stomach was the punctured site in all of the patients. Technical success was achieved in 96.67% (29/30) of the patients, and all of the patients who had EUS‐GJ 29/29 had clinical success (100%). Our results are comparable to other device‐assisted or EPASS techniques in terms of clinical or technical success, AEs, and median procedure time shown in Table 3.^12^, ^13^, ^14^ The majority of patients were able to eat without vomiting. The median procedure time in our study was 25 min. The median follow‐up in our study was 210 days, in previously published studies it varied from 66 to 319 days.^12^, ^13^, ^14^, ^15^ According to our study, the average length of hospitalization was 4.4 days. In previously published studies it varied from 2.2 to 12 days.^16^, ^17^, ^18^ In two prior studies, after EUS‐GJ, the median survival time was 103 days,^18^, ^19^ which is comparable to our study, which showed that EUS‐GJ can be effective in many patients with advanced malignant GOO. Stent misdeployment, bleeding, along obstruction recurrence are AEs associated with EUS‐GJ. In our study, only one patient (3.33%) had a stent misdeployment, and it was managed by the removal of the misdeployed stent and placement of an enteral SEMS. In previous studies rate of stent, misdeployment was 7%–36%.^1^, ^12^, ^18^, ^19^, ^20^, ^21^ None of our patients had post‐procedure bleeding or mortality. Procedure‐related peritonitis leading to the death of a patient has been reported by Tyberg et al.^20^ One of our patients had stent migration 10 months post‐procedure which was managed by the insertion of an enteral SEMS through the still patent GJ fistula.

**TABLE 3:** Comparison of nasojejunal tube‐assisted endoscopic ultrasound‐guided gastrojejunostomy (EUS‐GJ) with other techniques

**Technique**	**Author**	**Patients**	**Technical success (%)**	**Clinical success (%)**	**Procedure time (min)**	**Median follow‐up (Days)**	**Adverse events (%)**
Direct	Kerdsirichairat et al.^13^	57	93	89.5	39	196 (malignant) 319 (benign)	3.5
Balloon‐assisted	Chen et al.^14^	22	90.9	90.9	89.9	85	9
EPASS	Itoi et al.^12^	20	90	100	25.5	100	10
Nasojejunal‐assisted	Current study	30	96.67	100	25	210	6.67

Abbreviation: EPASS, endoscopic ultrasound‐guided double‐balloon occluded gastrojejunostomy bypass.

In this study, we typically used a 20‐mm lumen‐apposing metal stent (HOT Axios; Boston Scientific, Marlborough, MA, USA). Electrocautery is used to introduce the distal end of the stent delivery system through the posterior gastric wall. This delivery system eliminates the requirement for balloon catheter dilatation or endoscopic removal before stent implantation. The most difficult element of EUS‐GJ is puncturing the jejunum since it is so flexible and may be easily shifted away from the stomach. Though various techniques for EUS‐GE have been developed, the optimal technique is still unclear. The major drawback of the direct approach is the difficulty of needle puncture accessing the small bowel because the lumen is usually collapsed. In device‐assisted techniques, regarding which device should be used, there is no consensus among experts. The EPASS technique which some consider being robust and safe is still not commercially available. However, the nasojejunal tube is easily available and easy to place in the small bowel. The nasojejunal tube (14F) achieves adequate distension of the bowel in a short time using a water pump for fluid instillation and hence provides a suitable target for the electrocautery‐enhanced device for puncture. Results of the current study show that the use of a nasojejunal tube for EUS‐GJ is a safe and effective option for the commercially unavailable EPASS device. However, randomized studies comparing various techniques for performing EUS‐GJ are needed to establish the optimal technique.

The main implication of our study is that EUS‐GJ can be safely performed using a nasojejunal tube, which is easily available and cost‐effective.

Strengths of our study include firstly, this is the largest study to describe the use of nasojejunal tubes uniformly in all patients for EUS‐GJ. Secondly, patients were followed up regularly until death or last contact. Our study has certain limitations, which include a small sample size and a single‐center retrospective study even though we had a prospective database, which has certain inherent limitations, including heterogeneity among the patients. All the procedure was performed by an experienced endosonographer in a tertiary referral center and hence the results may not be generalized.

## CONCLUSION

In conclusion, EUS‐GJ using a nasojejunal tube is safe and effective. Technical and clinical success is comparable to other techniques.

## CONFLICT OF INTEREST

None.

## Supporting information


**Video S1** Video demonstrating nasojejunal tube placement followed by distension of bowel with saline, methylene blue and contrast and subsequent puncture of jejunum with cautery enhanced tip of the Hot Axios stent delivery device and finally deployment of the stent
